# Integrating artificial intelligence into veterinary education: student perspectives

**DOI:** 10.3389/fvets.2025.1641685

**Published:** 2025-08-04

**Authors:** Christelle de Brito, José I. Redondo, Irene Tadeo-Cervera, Esther Bataller, Mireia García-Roselló, Inmaculada Cuquerella Madoz, José Terrado

**Affiliations:** ^1^Departamento de Medicina y Cirugía Animal, Facultad de Veterinaria, Universidad Cardenal Herrera-CEU, CEU Universities, Valencia, Spain; ^2^Departamento de Producción y Sanidad Animal, Salud Pública Veterinaria y Ciencia y Tecnología de los Alimentos, Facultad de Veterinaria, Universidad Cardenal Herrera-CEU, CEU Universities, Valencia, Spain; ^3^Departamento de Humanidades, Facultad de Humanidades y Ciencias de la Comunicación, Universidad Cardenal Herrera-CEU, CEU Universities, Valencia, Spain

**Keywords:** artificial intelligence, higher education, perceptions, students, veterinary

## Abstract

**Introduction:**

Advancements in technology have fostered a continuous evolution of higher education, driving the adoption of innovative tools, including artificial intelligence (AI). This study explores veterinary students’ interest in AI, their training and experiences, and their perceptions on AI integration in veterinary medicine.

**Methods:**

A comprehensive survey was administered to veterinary students at the Faculty of Veterinary Medicine of a single international university in Spain, focusing on their experience with AI, their perception of its integration into veterinary education, and their views on its future role in veterinary medicine.

**Results:**

Six hundred and four students of 34 nationalities across all academic years answered the survey. Most students were familiar with AI tools and primarily utilize them in academic settings, recognizing AI as a valuable educational resource. The majority believed universities should encourage and regulate AI use. There was a strong desire to integrate AI-related education into the veterinary curriculum, with students eager to learn more about specific AI applications in various veterinary fields, in particular clinical patient monitoring and veterinary management. The study also highlights the need for training in AI principles and regulation. Likewise, students expressed concerns about ethical and responsible use of AI, as well as the reliability of AI responses.

**Discussion:**

This study underscores the importance of integrating AI training in veterinary education to enhance students’ competencies. By providing targeted training and support, universities can help students harness the potential of AI while ensuring its ethical and effective use in their careers. This research emphasizes the need for continuous curriculum adaptation to keep pace with technological advancements and meet the evolving demands of veterinary medicine education.

## 1 Introduction

In recent years, the rapid advancement of technology, coupled with the demand for new hybrid and asynchronous learning models driven by the COVID-19 pandemic, has led to the widespread adoption of innovative tools in the educational sector. These tools include digital learning platforms, learning management systems, online collaboration tools, educational applications, interactive whiteboards, extended (augmented and virtual) reality, as well as simulation and gamification tools, among others (1–5). These innovations have transformed teaching and learning processes, fostering a more digitized, interactive, personalized, and efficient learning environment (6).

Simultaneously, generative artificial intelligence (AI) has emerged as one of the most disruptive technologies in higher education as it can benefit both lecturers and students (7, 8). AI supports educators by helping with lesson planning, creating materials, and automating tasks and it also enhances student learning through personalized content, real-time feedback, interactive tools, and virtual simulations (9).

AI is now widely used across many professional fields, including human and veterinary medicine. The World Health Organization (WHO) published its first global report on AI in health in 2021 (10) and the World Medical Association (WMA) encourages the integration of AI in medical curricula (11). In human medicine, various studies suggest that AI can enhance patient care through applications such as precision medicine (personalized treatments through big data analysis), computer-assisted surgery, disease prediction and prevention (epidemiology, pharmacovigilance), therapeutic decision support, and remote monitoring (12–17). AI can also significantly impact veterinary fields by enhancing disease diagnosis, surveillance, treatment, surgery, drug and vaccine development, and cancer and antimicrobial resistance research (18). AI-based programs are being developed not only for clinical applications such as prevention, early detection of pathologies, diagnosis and decision support, but also for species conservation, animal welfare, optimization of animal production, and epidemiology (19–30). This broadening scope reflects a growing academic interest in the integration of AI into veterinary science, as evidenced by recent bibliometric analyses that has identified 1,400 published articles using the keywords “artificial intelligence” and “veterinary medicine,” the vast majority of which have been published since 2019, a year that marks the beginning of a rapid acceleration in publication rates (31). As AI has revolutionized both human and animal medicine, its integration into health science educational frameworks has also grown. Numerous studies have described the advantages of incorporating AI in the pedagogy of various health science disciplines. Examples include the application of ChatGPT to prepare clinical cases for problem-based learning strategies (32–34), the creation of virtual patients to develop diagnostic skills (35), and the provision of instant personalized medical information and monitoring of student learning. Recent studies have also highlighted the potential of using ChatGPT in veterinary education as it is a field less explored than human medicine that can, therefore, benefit from additional studies and a deeper analysis (36, 37). Notwithstanding these undeniable strengths, it is also important to mention weaknesses and ethical concerns of using AI in health science education (38). Studies in medicine highlight key limitations of AI, including possible lack of scientific accuracy, image recognition challenges, algorithmic bias, vague information definitions, and data privacy concerns (34, 39–42). Another disadvantage is the potential misuse of AI due to inadequate training, which can lead to errors in information resulting from incorrect prompts (43, 44).

The European Coordinating Committee on Veterinary Training (ECCVT) emphasized the importance of preparing the veterinary profession to face the challenges of digital technologies and AI through adequate and continuously updated training at the undergraduate, postgraduate, and continuing professional development levels (45). To address these challenges, it is crucial to discuss the necessity of adapting curricula to include AI-related activities. Understanding students’ perceptions of AI, their use of different tools, and their expectations is essential as a basis for planning possible actions that enable students to learn to use these tools effectively and ethically.

This study aims to explore these aspects through a comprehensive questionnaire conducted at an international Spanish Faculty of Veterinary Medicine and a combination of quantitative and qualitative analyses. The survey evaluated students’ interest in AI, their training and experiences, as well as their perceptions, expectations and vision of the use of AI in veterinary medicine. By gathering opinions from 604 students of 34 nationalities, with varying levels of education and ages, this study provides a valuable view of how veterinary students interact with AI tools, offers insights for curriculum development and contributes to the broader conversation on the responsible integration of AI in higher education.

## 2 Materials and methods

### 2.1 Approvals and ethical considerations

The study received approval by the Ethics Committee for Biomedical Research of the CEU Cardenal Herrera University (CEEI 24/554). Student’s participation in the survey was voluntary and anonymous. The study met the requirements of the Declaration of Helsinki. Participants could withdraw at any time, and their confidentiality was ensured as no personal information was collected. The data were available only for research.

### 2.2 Participants

This study examined students’ perceptions in the Faculty of Veterinary Medicine at the CEU Cardenal Herrera University during the 2024–2025 academic year, including students from all five academic years.

### 2.3 Survey

A multilingual survey was created using Microsoft Forms in Spanish, French, and English, ensuring comprehensive coverage across the cohort’s diverse academic and linguistic backgrounds. The survey was restricted to CEU Cardenal Herrera University members, with one response allowed per participant. The questionnaire was adapted from previously published studies (46–49). The questionnaire, originally with four sections and 36 questions, was refined through content, format, and translation reviews. Content validity was based on Lynn’s method (50). Face validation evaluated clarity, layout, and relevance and, as a result, the questionnaire was reorganized to 22 questions distributed in three sections: (1) general interest and demographic data, (2) AI integration in veterinary education and (3) student experience and future insights on AI in veterinary medicine (see Supplementary data 1).

Likert-scale questions generally included five levels, for instance: *strongly disagree*, *somewhat agree*, *neutral*, *somewhat agree*, *strongly agree*. In some case, the answer *I do not know* was also included.

The final questionnaire was distributed between November 26 and December 17, 2024, through a quick response (QR) code displayed during various teaching activities. Veterinary students were orally briefed on the study’s aims and methodology.

### 2.4 Data analysis

Statistical analyses were performed using Microsoft Excel and the R statistical language program (4.2.2) and several packages for data manipulation, visualisation, and modelling. After preparing the dataset and setting factor levels, descriptive statistics for demographic and categorical data were calculated. The descriptive analysis is presented as frequency table and charts. For Likert scale analysis each variable group was standardised for consistent levels. Given the nature of certain questions, the option “I do not know” was occasionally included in the questionnaire. Although the corresponding responses are presented in the results section due to their descriptive value, they were excluded from the statistical analyses as they reflected a lack of opinion or insufficient familiarity with the item rather than a position on the underlying construct. The “Likert” package for R visualised response distributions. Individual variables were plotted with bar charts showing response percentages for each category. Four groups were evaluated, each reflecting a distinct theme domain:

- Learning and faculty engagement: variables related to the perceived usefulness of AI in educational contexts and the role of faculty in promoting and regulating AI usage, including *I do not know*, *strongly disagree*, *somewhat disagree*, *neutral*, *somewhat agree*, to *strongly agree*.
- Knowledge and usage: variables that measure self-reported knowledge of AI principles and regulations, as well as the extent of AI integration into veterinary practice, including *I do not know*, *strongly disagree*, *somewhat disagree*, *neutral*, *somewhat agree*, to *strongly agree*.
- Satisfaction: variables capturing satisfaction with specific AI tools, such as text generation, image diagnosis, and clinical management aids, including *I do not know*, *strongly disagree*, *somewhat disagree*, *neutral*, *somewhat agree*, to *strongly agree*.
- Usage frequency: variables indicating the frequency of using AI tools, including *never, less than once a month, 1 to 3 times a month, 1 to 3 times a week and more than three times a week*.

In addition to descriptive statistics, several complementary multivariate techniques were applied to move beyond mere frequency counts and capture the latent structure of the data.

Spearman’s correlation analyses were performed for each thematic group and the entire dataset to examine the relationships among the survey variables. First, each set of Likert-scale variables was extracted and converted to numeric form for correlation analysis. The “cor” function was employed with the Spearman method to accommodate the ordinal nature of the data. Only complete cases were considered. The resulting correlation matrices were visualised via the “corrplot” package.

An exploratory factor analysis was conducted to extend the scope beyond correlation analysis using the “*fa*” function from the “psych” package. All Likert-scale variables were again converted to numeric values, and a three-factor solution with Varimax rotation was specified. This analysis was used to identify underlying dimensions of attitude that cannot be inferred from marginal distributions; this step ensured that subsequent interpretations rested on empirically derived constructs rather than on *ad hoc* thematic groupings.

Item response theory modelling used the “mirt” package. A unidimensional model was fitted to assess how well the individual items captured an underlying latent trait. This procedure provided insights into the discrimination and difficulty parameters of the survey items, helping to clarify which variables best differentiate between different levels of the latent trait in question.

Lastly, a network analysis was carried out to illustrate and explore the interrelationships among variables graphically, enabling the identification of central or bridging items that might play a pivotal role in knowledge translation or behavioural change. A thresholding procedure 0.3 was employed to include only the stronger correlations as edges in an adjacency matrix. The “igraph” package was then used to construct a directed network, and the Fruchterman–Reingold algorithm was specified to position the nodes.

Collectively, these approaches offer insights into the coherence, reliability and interdependence of students perceptions of AI that cannot be obtained from descriptive statistics alone.

To conduct analysis of open-ended questions, all responses provided by veterinary students were translated into English and the most frequently elements were identified. Then elements were categorized, and specific notions were noted. The information was synthesized to identify general trends. Word clouds were generated in April 2025 with Copilot to visually represent the frequency of terms mentioned in the response. The size of each word is representative of its frequency of occurrence in the open responses.

## 3 Results

### 3.1 Students’ general interest in AI and demographic data

**Figure 1:**
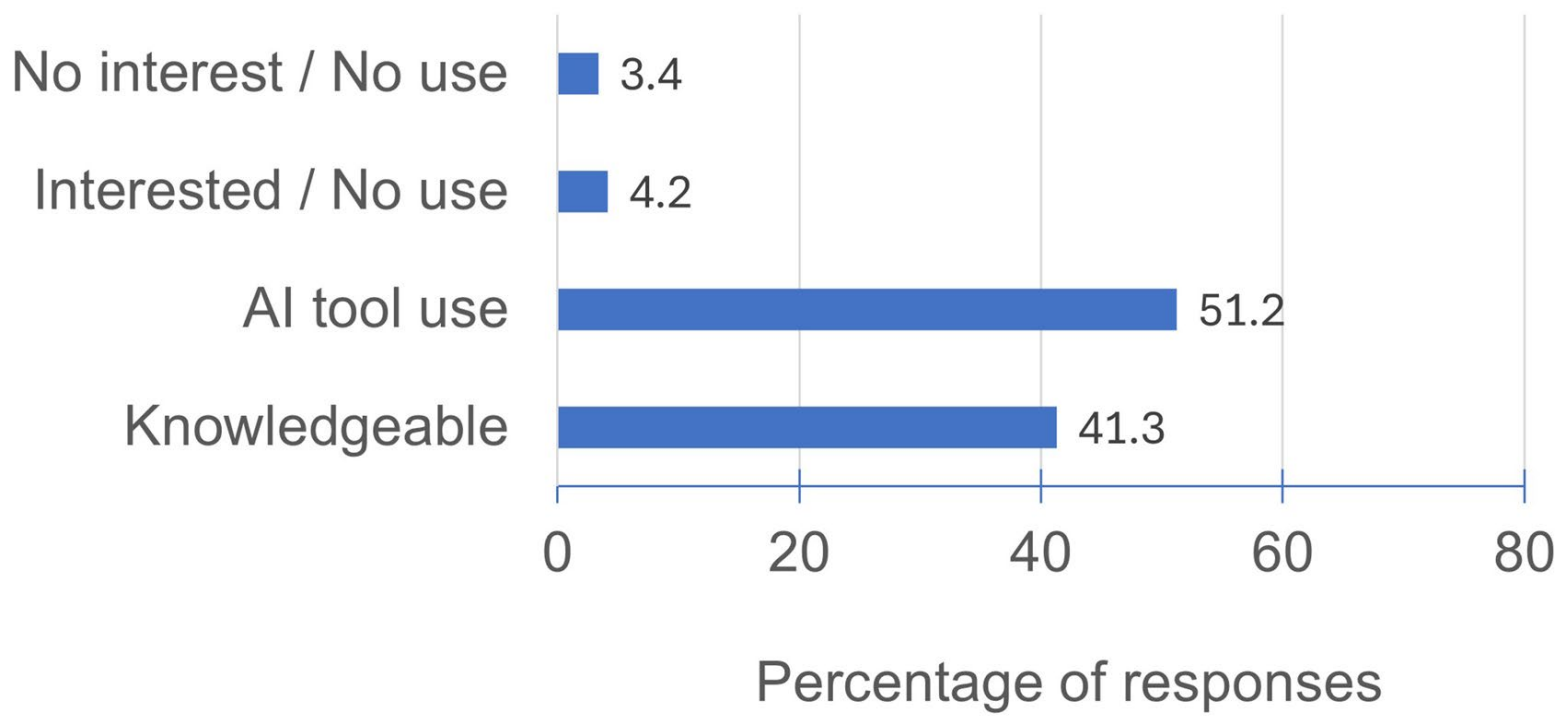
Respondents’ attitude towards AI. The bar chart illustrates the distribution of responses regarding students’ familiarity and interest in AI. Legends of the responses: No Interest/No Use: “I have never used AI-assisted tools and I have no interest in AI in general;” Interested/No Use: “I am interested in AI but I have never used AI-assisted tools;” AI tool use: “I have used an AI-assisted tool at least once in my life;” Knowledgeable: “I believe I have knowledge about the strengths and weaknesses of AI-assisted tools”.

Participants from all academic years at the Faculty of Veterinary Medicine were recruited during academic activities throughout the five-year curriculum (Table 1). Six hundred and twenty-five students (38.5% of the total enrolled students) participated. Most participants demonstrated familiarity with AI (Figure 1), either by having used AI at least once (51.2%) or by understanding its strengths and limitations (41.3%). However, 21 students (3.4% of the respondents) reported no familiarity with or interest in AI. Notably, most respondents who said they were not familiar with or interested in AI (72.2%) were first-year students. They were excluded from further analysis. Consequently, the final sample for subsequent analyses comprised 604 students.

**Table 1:** Survey response rates by academic year.

Academic year	Number of enrolled students	Number of responding students	Responding rate for each academic year (%)
1st	357	269	75.3
2nd	298	151	50.7
3rd	285	89	31.2
4th	302	67	22.2
5th	382	49	12.8
All	1,624	625	38.5

The mean age of the respondents was 20.5 years. Most students (84.5%) were between 18 and 22 (Table 2). The participants were predominantly female (82.8%). Most participants identified as Spanish (47.2%), followed by French nationals (43.4%), with the remaining 9.4% coming from 32 different countries, reflecting the international diversity of the students participating in the study. Most respondents (58.4%) reported having no prior higher education experience before starting their veterinary studies. Those who had pursued higher education mainly studied science-related fields. Most participants (68.7%) preferred clinical practice in the veterinary specialty.

**Table 2:** Demographic characteristics of students who completed the survey, number of responses obtained and corresponding percentages of the total respondents (*n* = 604).

Demographics	Number of respondents	Percentage (%)
Age 18 19 20 21 22 23 >23 No data	142 110 72 112 75 33 58 2	23.5 18.2 11.9 18.5 12.4 5.5 9.60.33
Gender Female Male Prefer not to say	500 102 2	82.8 16.9 0.3
Nationality Spanish French Other	285 262 57	47.2 43.4 9.4
Prior higher education None Sciences Technician Engineer Others	353 145 47 8 51	58.4 24.0 7.8 1.3 8.4
Veterinary specialty preference Clinical practice Animal production Conservation Public Health Other	415 73 68 21 27	68.7 12.1 11.3 3.5 4.5

### 3.2 AI integration in veterinary education

**Figure 2:**
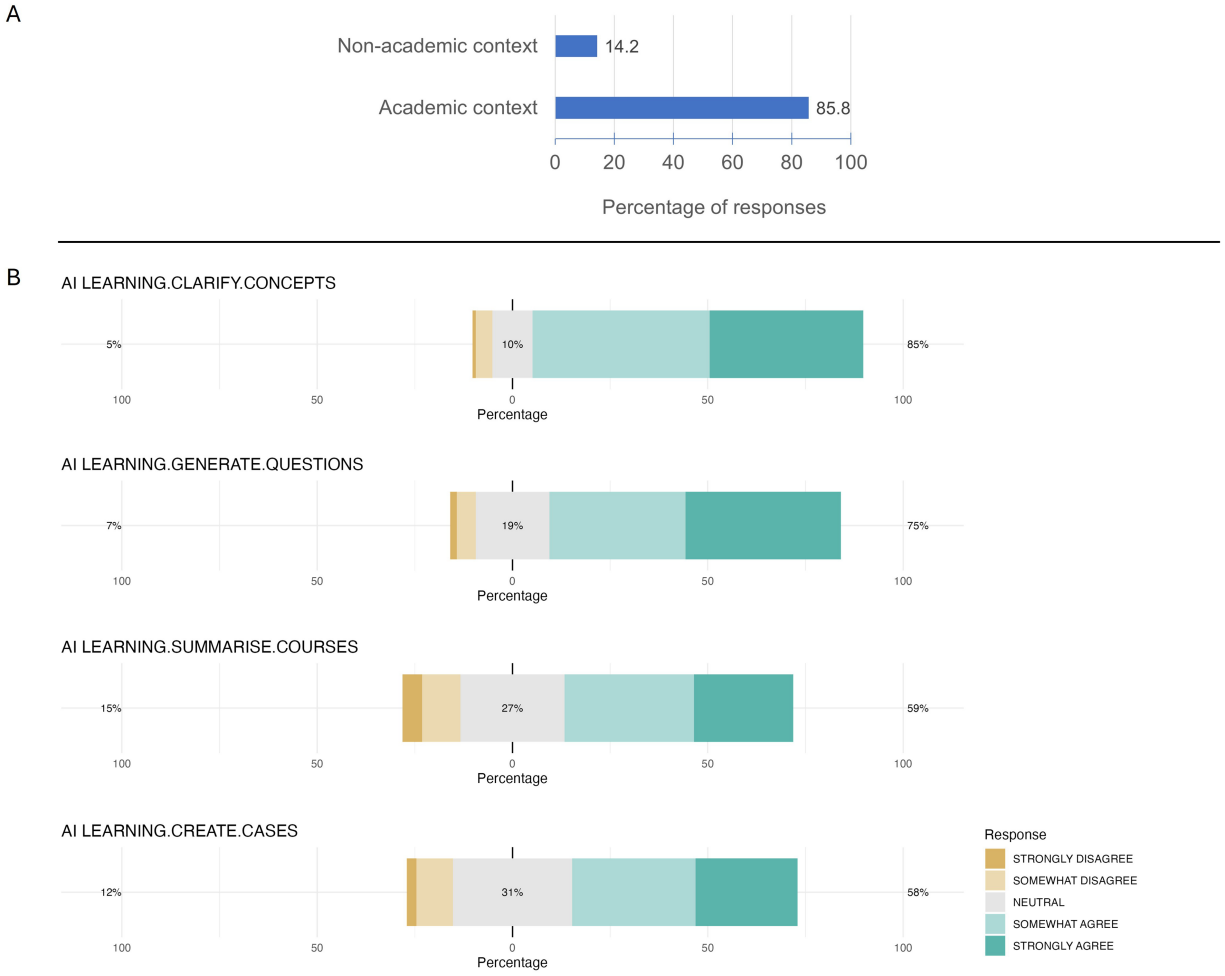
Context of AI tool usage and perceived utility for learning. **(A)** The bar chart illustrates the distribution of respondents’ AI tool usage contexts. The response options were: academic context and non-academic context. **(B)** The chart shows the distribution of respondents’ levels of agreement with various statements regarding the use of AI in veterinary medicine courses. The following statements were evaluated: AI facilitates obtaining clear explanations about complex concepts (AI LEARNING.CLARIFY.CONCEPTS), AI generates questions that help with exam preparation (AI LEARNING.GENERATE.QUESTIONS), AI allows summarizing veterinary medicine courses (AI LEARNING.SUMMARISE.COURSES), and AI allows generating clinical cases and patient simulations (AI LEARNING.CREATE.CASES).

Most students (85.8%) reported using AI tools more frequently in academic contexts than in other settings, and consistently, results show that AI is generally perceived by students as a valuable educational tool (Figure 2). The proportion of students who selected “I do not know” in response to questions assessing whether AI helped clarify veterinary concepts, generate exam preparation questions or summarize lectures remained low, at 1.8, 11.3, and 10.6%, respectively. Most participants who formed an opinion agreed that AI helped clarify veterinary concepts (84.7%), generate exam preparation questions (74.6%), and summarize lectures (58.5%). However, the percentage of students who answered “I do not know” when asked whether AI can generate clinical cases and patient simulations was higher (25.7%), while 57.7% of the students who expressed an opinion agreed with the statement.

**Figure 3:**
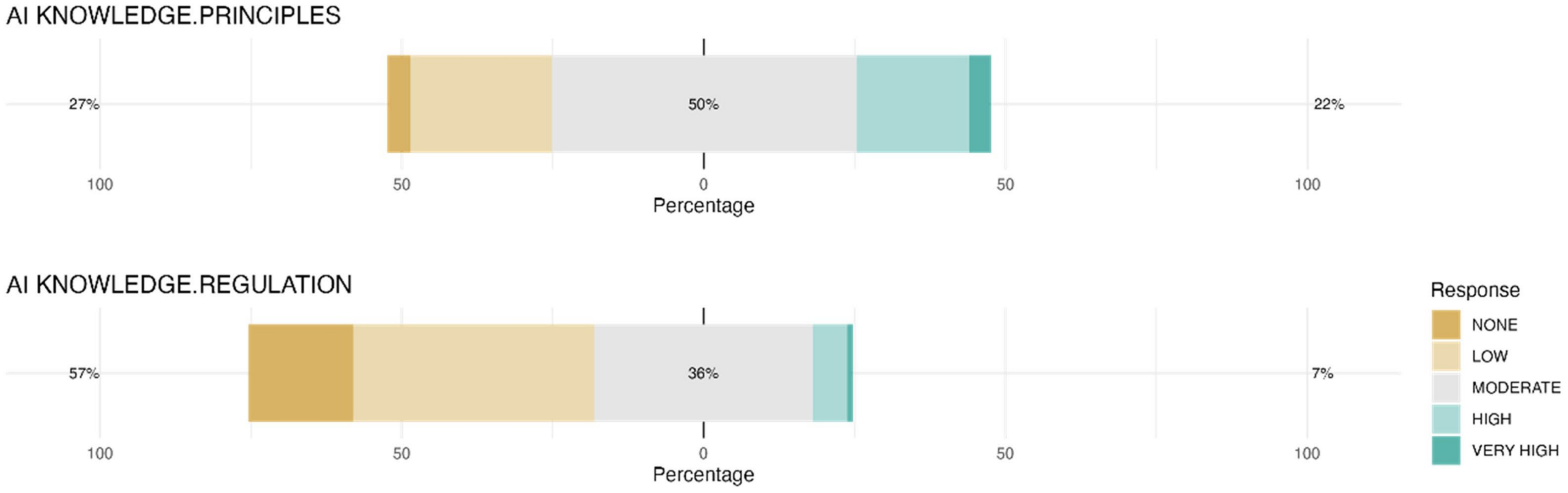
Students’ perception of their knowledge in AI principles and regulation. The chart illustrates the distribution of respondents’ self-assessed proficiency levels regarding the principles of AI operation (AI KNOWLEDGE.PRINCIPLES) and the regulation of AI (AI KNOWLEDGE.REGULATION).

The survey results showed important variability in student perceptions of their knowledge about fundamental AI principles. Most students rated their knowledge as moderate (50.5%), while fewer rated it as low or non-existent (27.1%), and even fewer rated it as high or very high (22.3%) (Figure 3). In contrast awareness of AI regulations was more limited, with most students (57.3%) considering their knowledge as low or nonexistent, suggesting that regulatory aspects may require additional attention in future training.

**Figure 4:**
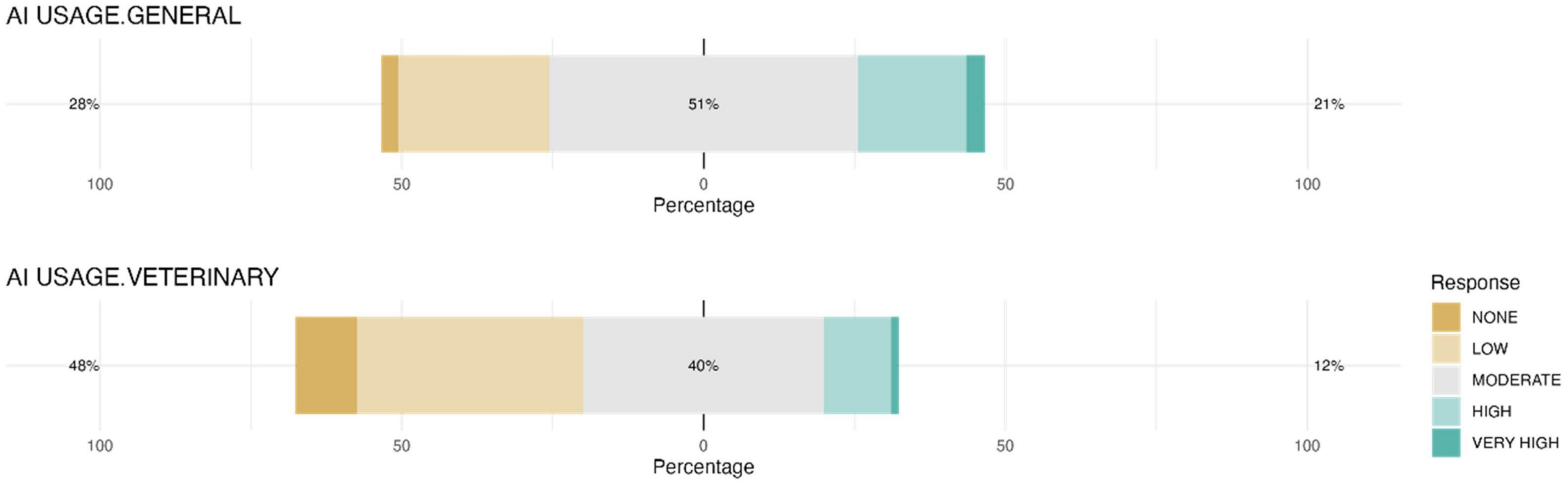
Students’ use of general and veterinary AI tools. The chart illustrates the distribution of respondents’ usage of general AI tools (AI USAGE.GENERAL) and AI tools that are specific to veterinary field (AI USAGE.VETERINARY).

Most students (51.2%) considered their use of general AI tools to be moderate (Figure 4). However, use of AI tools specific to veterinary medicine was lower, with most students (47.7%) reporting low or no use.

**Figure 5:**
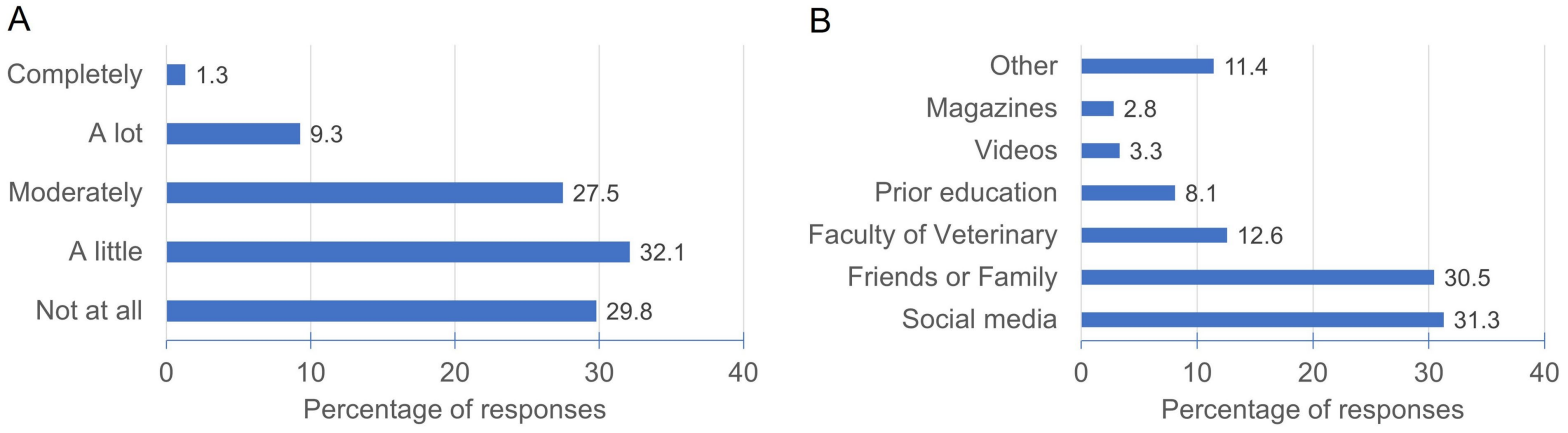
Role of the faculty in AI skill acquisition and major information sources. **(A)** The bar chart presents student perceptions of the extent to which teaching at the Faculty of Veterinary Medicine has contributed to their AI skill acquisition. **(B)** The chart illustrates the primary sources from which veterinary students obtained practical information about AI.

**Figure 6:**
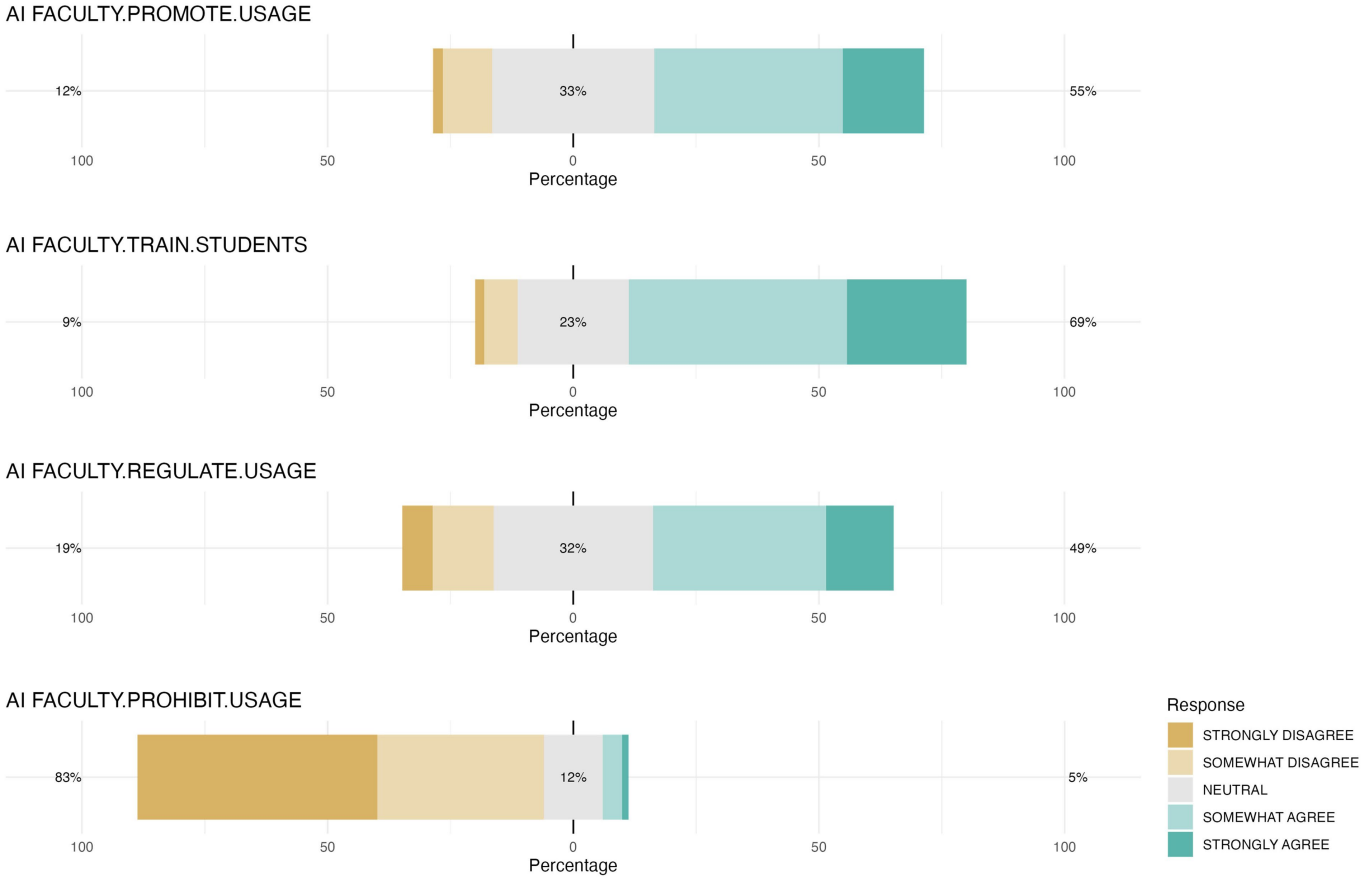
Student perceptions of faculty’s role in student AI use during education. The chart illustrates respondents’ levels of agreement with various statements regarding the stance that veterinary faculties should take on the use of AI in veterinary education. The statements include encouragement (AI FACULTY.PROMOTE.USAGE), training (AI FACULTY.TRAIN.STUDENTS), regulation (AI FACULTY.REGULATE.USAGE), and prohibition (AI FACULTY.PROHIBIT.USAGE) of AI use during veterinary training.

**Figure 7:**
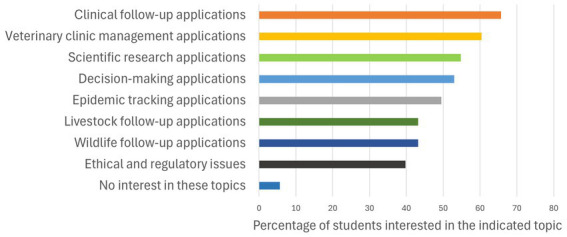
Preferred AI topics for veterinary training. The chart illustrates aspects of AI that veterinary students would like to cover during their veterinary training at the faculty. Multiple responses were allowed.

Responses about faculty’s role in AI skills acquisition revealed that most students perceive that teaching somewhat contributed to their AI-related competencies, with the highest percentage of students (32.1%) feeling that the faculty contributed a little, followed closely by those who felt that the faculty had not contributed at all (29.8%) and those who felt that it had contributed moderately (27.5%) (Figure 5). Consistently, only 12.6% of the students reported that the faculty was the primary source of practical information about AI, while the majority (61.7%) indicated they obtained this information through friends, family and social networks. The survey also indicated that an important portion of students believed veterinary faculties should promote the use of AI (55%), provide training in AI (68.7%), and regulate the use of AI by students (49%). In contrast there was strong opposition (82.7%) to the prohibition of AI use in the academic context (Figure 6). Furthermore, and in line with the general support for faculty encouragement of AI use, when students were asked which aspects of AI they would like to be included in the veterinary training, all proposed topics were deemed interesting, with 39.7 to 65.7% of students expressing interest in each (Figure 7). Notably, the highest interest was in clinical patient follow-up (65.7%) and veterinary clinical management (60.4%) applications, aligning with most of the students’ preference for clinical practice.

### 3.3 Student experience and future insights on AI in veterinary medicine

**Figure 8:**
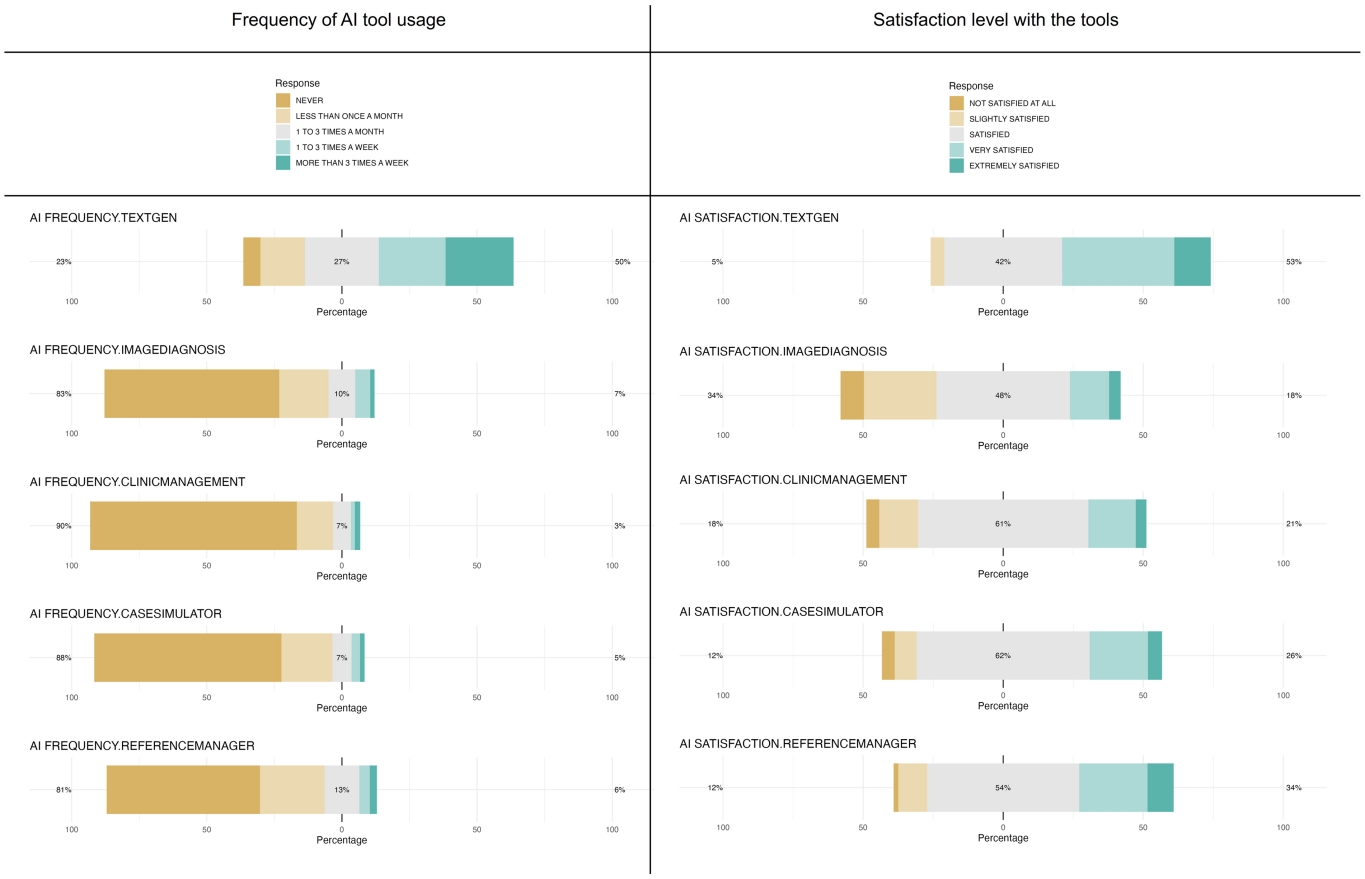
Frequency of AI tool usage by veterinary students and satisfaction levels with the tools. Left: the chart shows the frequency with which veterinary students reported to use AI tools enabling text generation (ChatGPT, Copilot, etc.), image analysis for diagnosis, veterinary clinic management, clinical case simulation and bibliographic reference assistance (Zotero, Mendeley, etc.), ranging from never to more than three times a week. Right: the figure shows the satisfaction levels of veterinary students with the same tools, ranging from “not satisfied” to “extremely satisfied”.

Students reported that they frequently use text generating AI, with half of the students (49.9%) using it on a weekly basis. The proportion of students who did not express an opinion to the question assessing satisfaction was low (7.9%) and the majority of students who provided an opinion indicated they were satisfied, very satisfied or extremely satisfied (95.0%). In contrast, most students stated they never used four kinds of AI tools: tools for imaging diagnostics (64.7%), clinical management (76.5%), case simulation (69.2%), and reference manager assistance (56.8%). In alignment with this data, over 60% of students indicated they did not know enough about these tools to provide an opinion (68.0, 78.5, 70.5, and 61.3% respectively). Among those who responded, more than 65% were satisfied, very satisfied or extremely satisfied with these four types of tools (65.9, 81.4, 87.5, and 87.9% respectively) (Figure 8).

**Figure 9:**
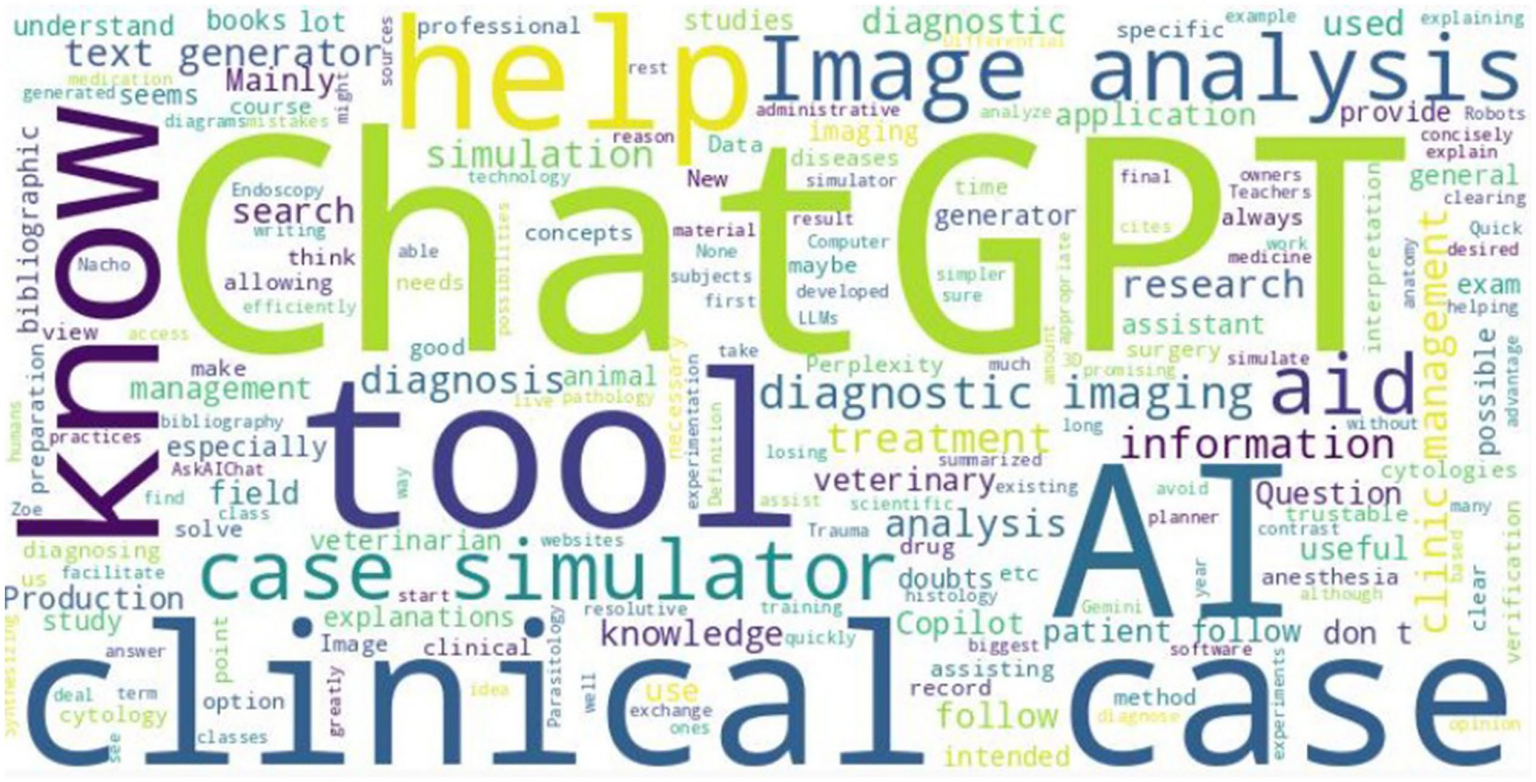
Promising aspects of AI identified by veterinary students. The largest words represent the most common terms used by veterinary students.

The analysis of the survey’s open responses regarding promising aspects related with AI reveals that veterinary students identified several promising AI applications in education and practice, with ChatGPT being the most frequently mentioned tool for its usefulness in studying and information synthesis. Students also valued image analysis for diagnostics, clinical simulators for hands-on training, and AI tools for clinic management. However, some expressed limited knowledge of veterinary-specific AI tools and concerns about their effectiveness (Figure 9).

**Figure 10:**
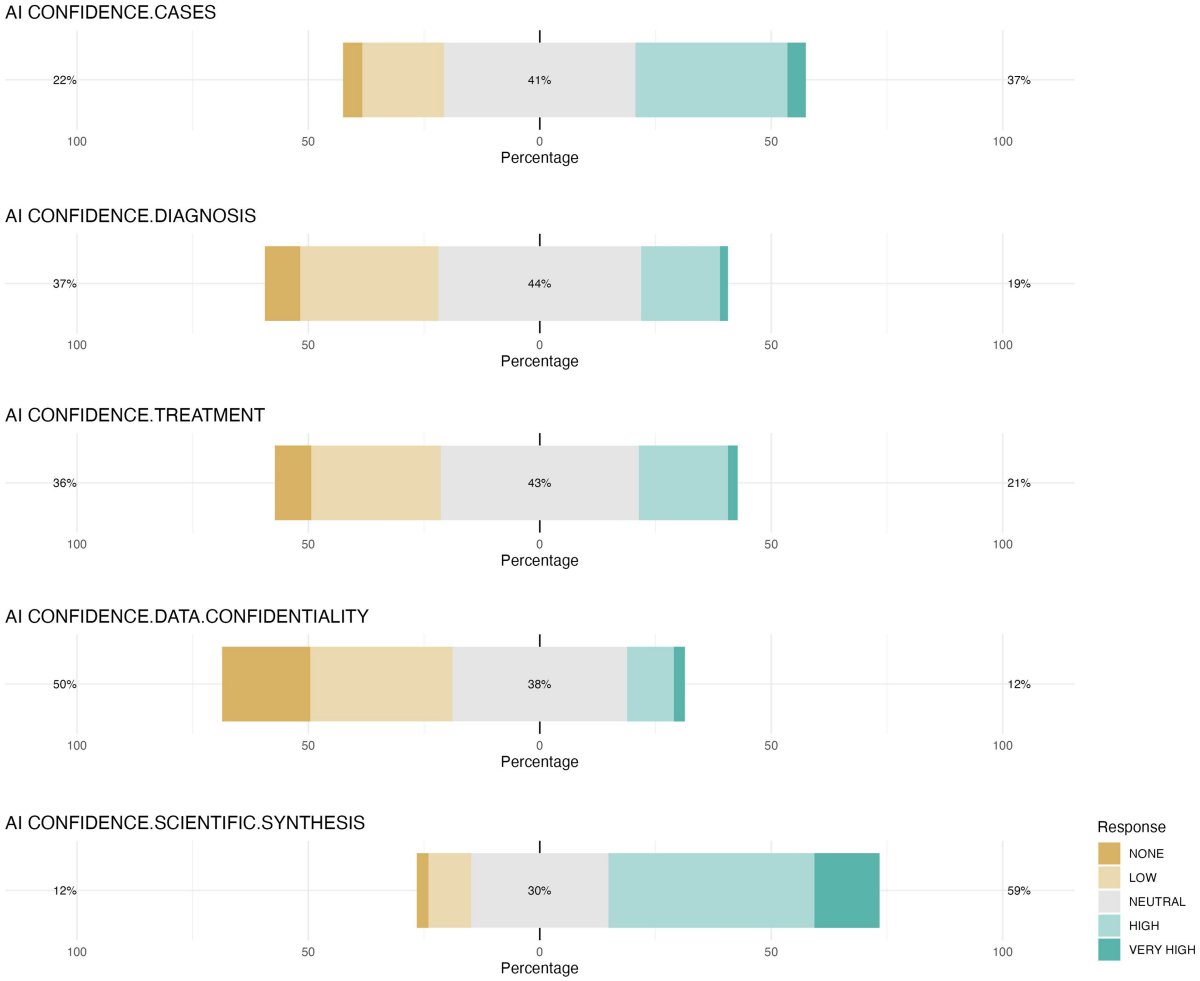
Confidence levels regarding various AI-generated elements. The figure shows the distribution of confidence levels among veterinary students concerning different aspects of AI-generated data including clinical cases generated by AI (AI CONFIDENCE.CASES), veterinary diagnosis established by AI (AI CONFIDENCE.DIAGNOSIS), veterinary treatment recommended by AI (AI CONFIDENCE.TREATMENT), confidentiality of personal data treatment by AI (AI CONFIDENCE.DATA.CONFIDENTIALITY), and synthesis of scientific information by AI (AI CONFIDENCE.SCIENTIFIC.SYNTHESIS).

Students exhibited a cautious perspective regarding AI capabilities across various veterinary-related fields. Results indicate that a notable percentage of students (over 20%) selected “I do not know” when asked about AI applications in veterinary education, with 24.8% for clinical case generation, 21.4% for diagnosis, 20.2% for treatment recommendations, and 24.5% for data privacy. Among those who provided an opinion, a neutral level of confidence was the predominant response, with approximately 40% of participants expressing neutral confidence (41.4, 43.8, 42.7, and 37.7%, respectively). The only item where positive opinions outweighed negative ones was confidence in the synthesis of scientific information by AI, with only 15.2% of the students expressing no opinion (Figure 10).

**Figure 11:**
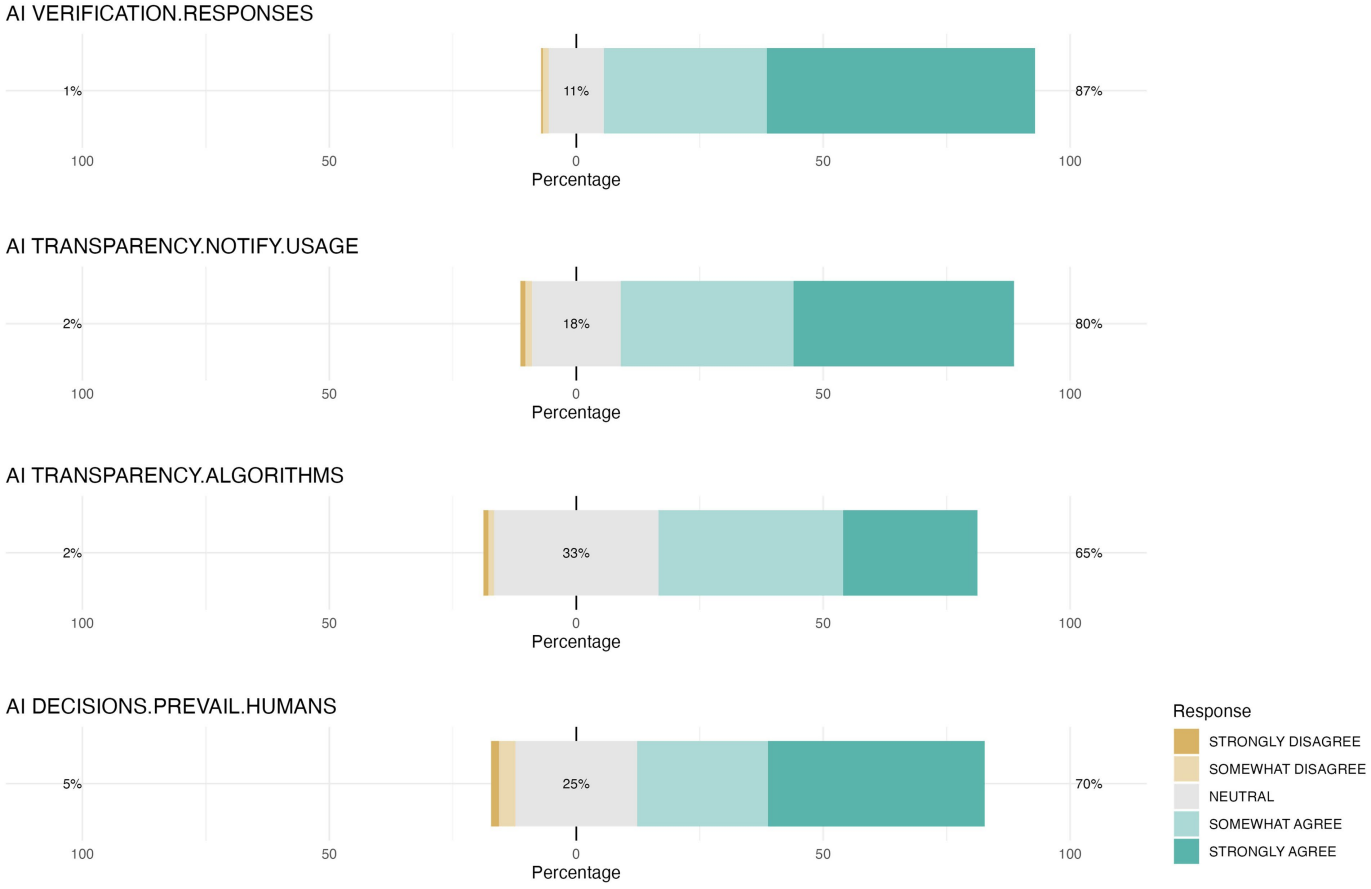
Students’ agreement levels on verification, transparency, and human oversight of AI-assisted tools. This figure illustrates the distribution of student agreement with statements about the necessity of verifying AI-generated responses (AI VERIFICATION.RESPONSES), the desire for transparency when using AI-assisted tools (AI TRANSPARENCY.NOTIFY.USAGE) and for information on how AI tools function, in particular, information about the algorithms that are used (AI TRANSPARENCY.ALGORITHMS), as well as opinion about prevalence of human decisions over AI-assisted decisions (AI DECISIONS.PREVAIL.HUMAN).

The survey also assessed aspects related to opinions about the need for verification of the information provided by AI systems and the transparency of AI participation in certain interventions. A large majority of students believed that AI-generated responses should be verified, with 54.3% strongly agreeing and 32.9% somewhat agreeing. Results about the perceived need of transparency in relationship to AI usage were similar, with 44.7% strongly agreeing and 34.9% somewhat agreeing that AI usage should be disclosed. Likewise, 37.4% somewhat agreed and 27.2% strongly agreed that algorithmic operations should be transparent. Additionally, a notable portion of students strongly agreed (43.9%) or somewhat agreed (26.5%) that human decisions should prevail over AI (Figure 11).

**Figure 12:**
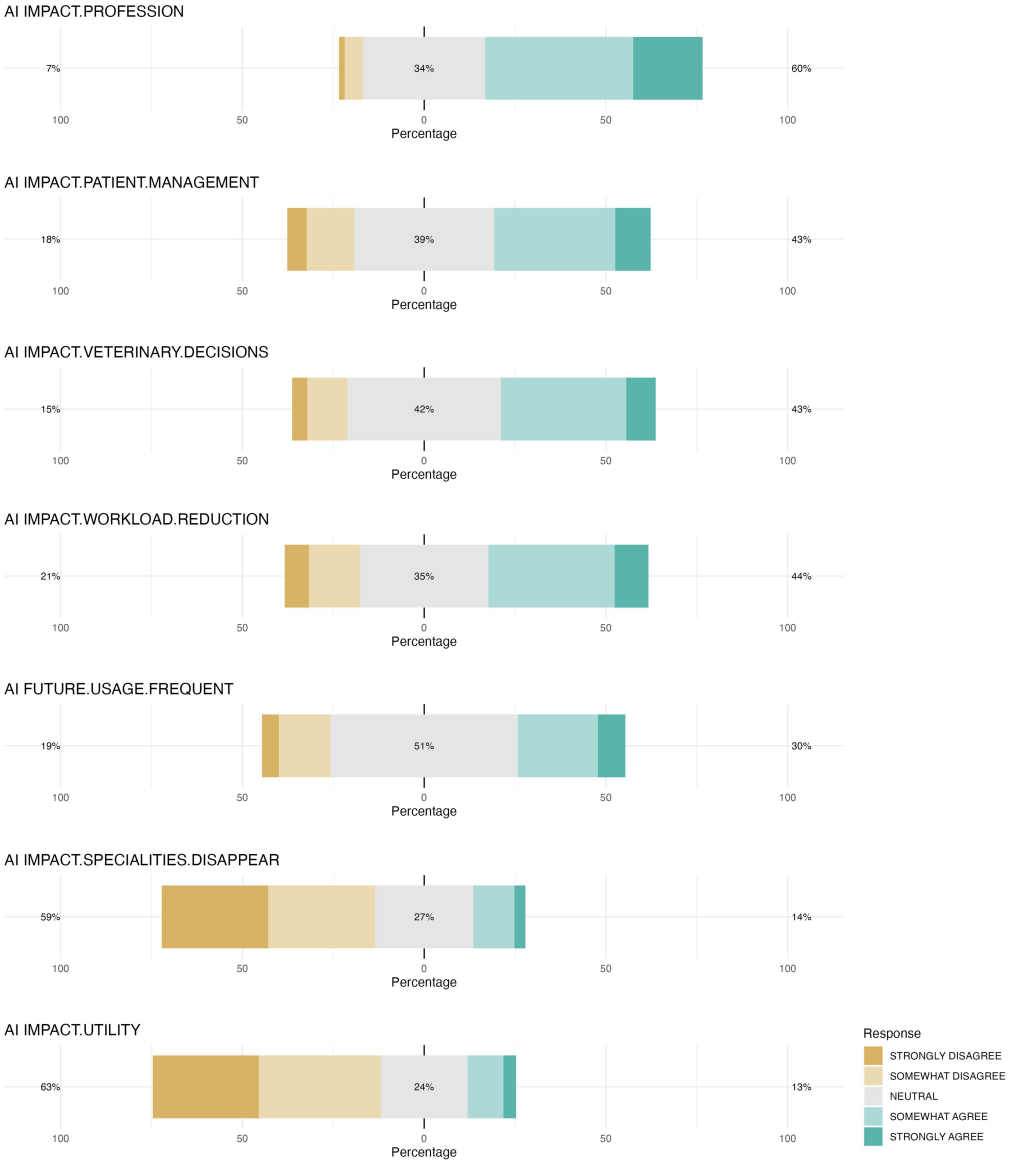
Distribution of veterinary students’ Likert scale responses on AI impact on the profession. The figure illustrates distribution of student responses regarding various statements, including AI will have a great impact on the veterinary profession (AI IMPACT.PROFESSION), AI will improve the care of animal patients (AI IMPACT.PATIENT.MANAGEMENT), will improve decisions made by veterinarians (AI IMPACT.VETERINARY.DECISIONS), AI will reduce the workload of veterinarians (AI IMPACT.WORKLOAD.REDUCTION), when I become a veterinarian, I will frequently use AI-assisted tools (AI FUTURE.USAGE.FREQUENT), AI will make some veterinary specialties disappear (AI IMPACT.SPECIALTIES.DISAPPEAR), AI will decrease the usefulness of veterinarians (AI IMPACT.UTILITY).

Students’ Likert scale responses regarding AI’s impact on the veterinary profession, including its overall influence, improvement of animal care and decision-making, as well as reduction of workload, exhibited a similar pattern. Most students were either neutral (approximately 37%) or somewhat agreed (approximately 35%) with these statements. Similarly, the responses to the statement about future personal use of AI tools showed a comparable trend, with a slightly higher percentage of neutrality (51%). In contrast, a different response pattern emerged for the statement that AI could potentially lead to the disappearance of certain veterinary specialties or diminish the utility of veterinarians. These notions were generally rejected, with around 30% of students strongly disagreeing and another 30% disagreeing (Figure 12).

**Figure 13:**
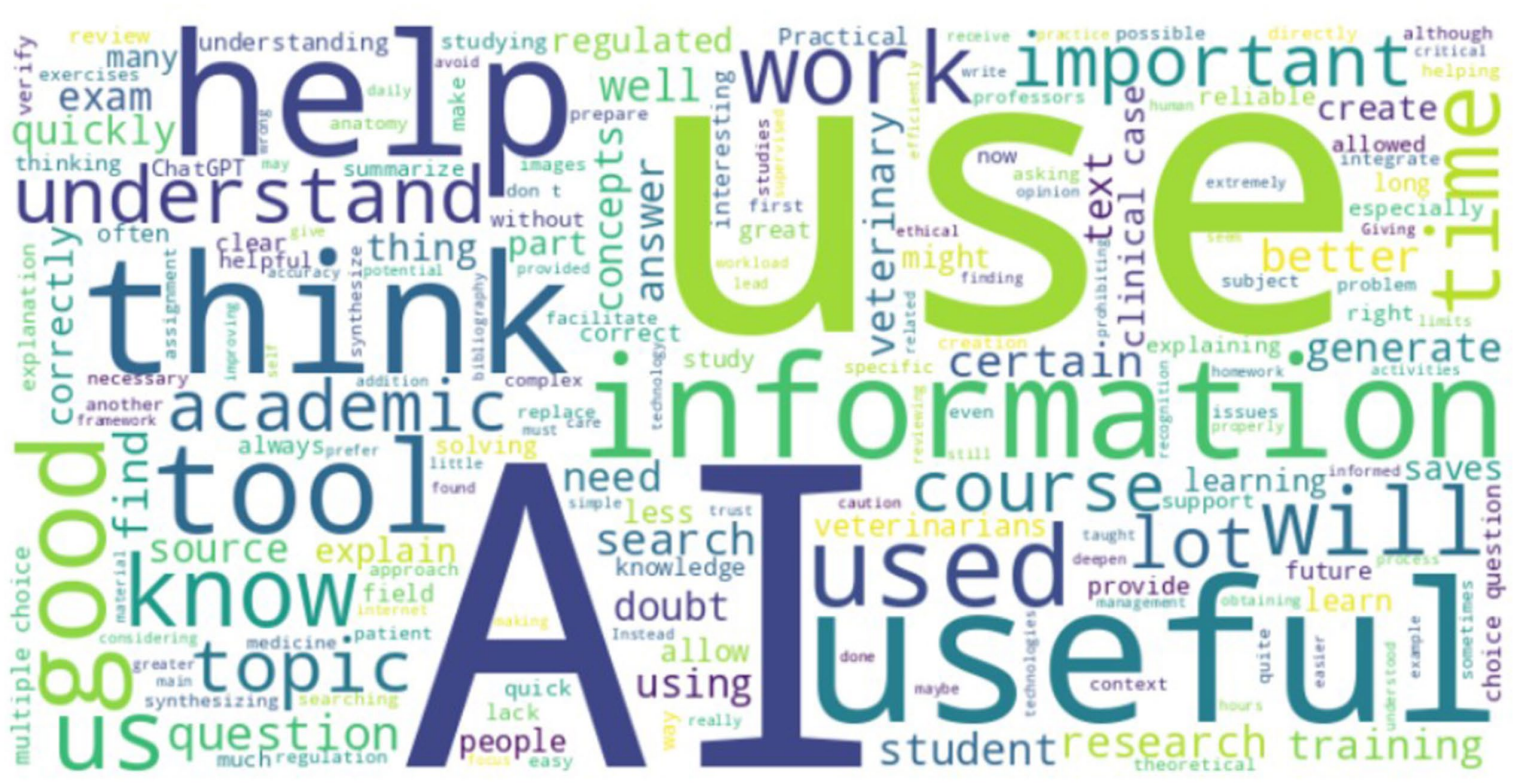
Students’ final comments about use of AI in academic context. The largest words represent the most common terms used by veterinary students.

At the end of the survey, students were invited to share their perspectives on the use of AI in academic activities through a final open-ended question. Many students viewed AI as a valuable support tool, especially for information search, summarizing content, and literature reviews. It was recognized for saving time, improving task efficiency, and aiding in study and exam preparation. However, concerns were raised about the need for regulation, potential misuse, reliability issues, and its impact on critical thinking (Figure 13).

### 3.4 Correlation analysis

**Figure 14:**
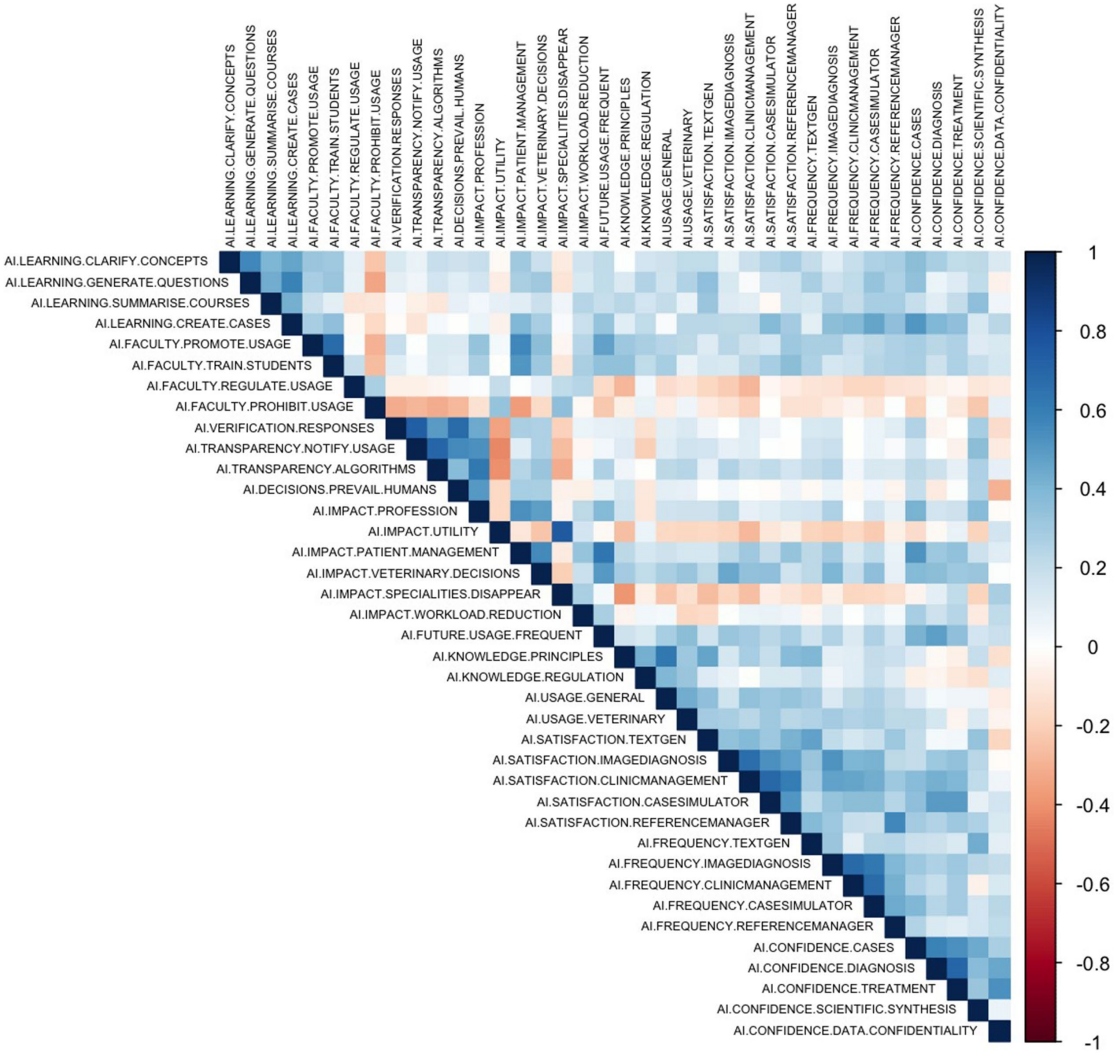
Correlation heatmap of student perceptions on IA use in veterinary education. This heatmap illustrates Spearman’s correlation coefficients among survey variables of the study. The color ranges from −1 (dark red) to 1 (dark blue), indicating the strength and direction of the correlations.

The correlation matrices visualization aided in identifying meaningful associations within thematic groups (Figure 14). Table 3 presents the item pairs whose correlation coefficients exceed 0.70, indicating strong relationships, all statistically significant at *p* < 0.001 following Bonferroni correction for multiple comparisons. The highest correlation (*ρ* = 0.76) was observed between the belief that AI may lead to the disappearance of certain veterinary specialities and the perceived impact of AI on the future utility of veterinarians. This association reflects a coherent perception of AI’s disruptive potential among respondents. A similarly strong correlation (*ρ* = 0.73) was found between the perceived need to verify AI-generated responses and the perceived importance of being informed when a tool is AI-assisted, indicating a consistent concern for transparency and accountability. This theme was further supported by the correlation between transparency regarding how AI-assisted tools work, particularly the algorithms they use, and the notification of AI use (*ρ* = 0.71). Other notable associations included the relationship between confidence in AI-assisted diagnosis and treatment (*ρ* = 0.70) and between satisfaction with the clinical case simulator and clinic-management tools (*ρ* = 0.70). While these findings indicate areas of conceptual convergence, no evidence of redundancy was observed. Each item contributed unique variance to the overall construct, supporting the multidimensional nature of students’ engagement with and attitudes toward AI.

**Table 3:** Item pairs with strong positive correlations.

Variable 1	Variable 2	Correlation coefficient
AI.IMPACT.SPECIALTIES.DISAPPEAR	AI.IMPACT.UTILITY	0.76
AI.TRANSPARENCY.NOTIFY.USAGE	AI.VERIFICATION.RESPONSES	0.73
AI.TRANSPARENCY.ALGORITHMS	AI.TRANSPARENCY.NOTIFY.USAGE	0.71
AI.CONFIDENCE.TREATMENT	AI.CONFIDENCE.DIAGNOSIS	0.70
AI.SATISFACTION.CASESIMULATOR	AI.SATISFACTION.CLINICMANAGEMENT	0.70

### 3.5 Exploratory factor analysis

Before conducting the exploratory factor analysis, the data were assessed for suitability. The Kaiser–Meyer–Olkin (KMO) measure of sampling adequacy was 0.88, indicating meritorious sampling adequacy, and Bartlett’s test of sphericity was highly significant [*χ*^2^(703) = 9,173, *p* < 0.001], confirming that the correlation matrix was appropriate for factor extraction. The analysis of the survey responses showed that the questions naturally grouped into three main factors or themes, and this structure was consistent and reliable.

These three factors explained about 28% of the variance [sum of square loadings (SS loadings) = 4.43, 3.38, and 2.94 for factors 1–3] and therefore of the differences in how students answered the questions, which is considered acceptable in this type of research. Fit indices showed acceptable, though improvable, performance: Likelihood *χ*^2^(592) = 4,506, *p* < 0.001; root mean square error of approximation (RMSEA) = 0.10 (90% CI 0.10–0.11); root mean square of the residuals (RMSR) = 0.09; Tucker Lewis index of factor reliability (TLI) = 0.45; Bayesian information criterion (BIC) = 715. The first theme was confidence in using AI with 10 items loaded ≥0.40. Factor-loading patterns reproduced the thematic structure previously described. Eight items loaded ≥0.40 on the “Confidence in AI” factor, eight on “Education and faculty support,” and five on “Use and satisfaction with AI tools,” supporting content validity while suggesting that further refinement (e.g., through confirmatory factor analysis) could improve model fit. Overall, these results confirm that the survey effectively measures three separate but important areas, each related to a key part of the study’s goals.

### 3.6 Item response theory

A unidimensional graded-response item response theory model converged satisfactorily after 21 iterations, yielding a final log-likelihood of −23975.71. Discrimination parameters (a) ranged from 0.12 to 0.66, indicating that all items contribute to differentiating respondents along the latent attitude continuum. The strongest discrimination was observed for “Future usage (frequent)” (*a* = 0.66) and “Confidence in diagnosis” (*a* = 0.65). In contrast, the lowest values were seen for “Transparency: notify usage” (*a* = 0.12) and “Decisions prevail humans” (*a* = −0.09). Communalities (*h*^2^) spanned from 0.01 to 0.44, confirming that most items share meaningful common variance with the underlying trait. These results demonstrate that, overall, the questionnaire reliably captures variation in students’ engagement with and perceptions of AI.

### 3.7 Network analysis

**Figure 15:**
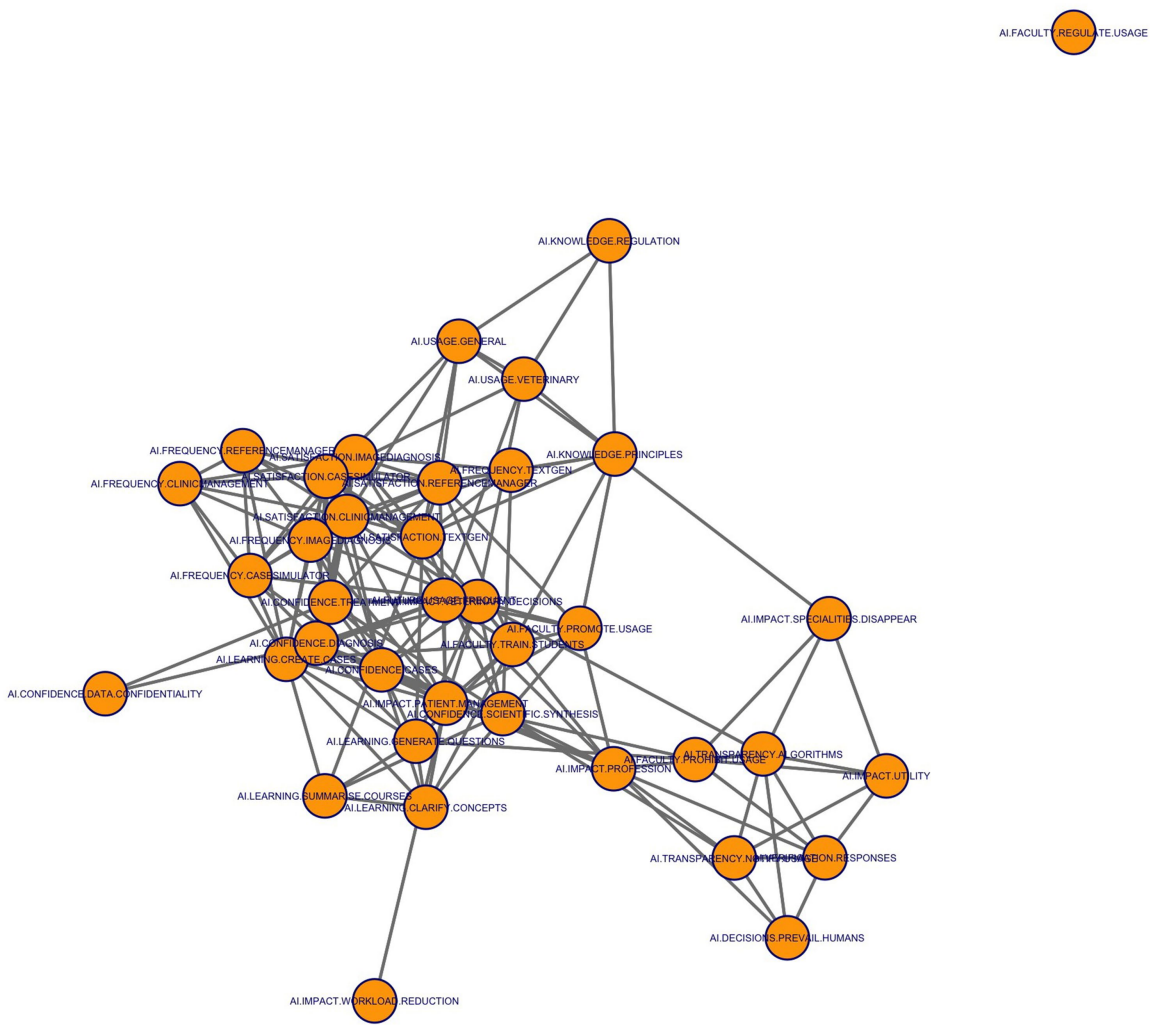
Network analysis of AI engagement factors among veterinary students. The figure presents the correlation network from 37 questionnaire items (*n* = 604), where nodes represent key themes and edges indicate conceptual relationships based on co-occurrence or similarity. Clusters highlight groups of closely related concepts.

Figure 15 provides a visual depiction of the inter-item correlation network (edges shown for |*ρ*| ≥ 0.50). Nearly all questionnaire statements are part of a single, densely connected cluster: 36 of the 37 nodes group together into one broad assembly, leaving only the statement about the need of regulation by the faculty (AI.FACULTY.REGULATE.USAGE) disconnected from its peers. Its separation suggests either conceptual uniqueness or a limited response range that sets it apart from the common discourse on AI. Within the main cluster, attention is drawn to a tight core where items related to frequency of use and confidence form a highly interconnected web. Statements on clinical confidence (e.g., AI.CONFIDENCE.DIAGNOSIS, AI.CONFIDENCE.TREATMENT), frequency of use (AI.FREQUENCY.CASESIMULATOR, AI.FREQUENCY.CLINICMANAGEMENT), and educational benefits (AI.LEARNING.CREATE.CASES, AI.KNOWLEDGE.PRINCIPLES) sit at the centre of this network knot. Their high betweenness scores indicate they serve as bridges, facilitating communication between otherwise separate thematic areas. A second, clearly identifiable pattern appears along the lower-right side, where items related to transparency and verification (AI.TRANSPARENCY.NOTIFY.USAGE, AI.VERIFICATION.RESPONSES, AI.TRANSPARENCY.ALGORITHMS) group together, sharing strong bilateral links but connecting back to the core through a few key ties. Finally, a more peripheral strand connects perceptions of AI’s professional consequences: AI.IMPACT.UTILITY, AI.IMPACT.SPECIALITIES.DISAPPEAR, and AI.DECISIONS.PREVAIL.HUMANS form a triple arc extending away from the centre, implying that views on AI’s disruptive potential, though related to the main discussion, occupy a somewhat separate conceptual space.

## 4 Discussion

This study explores the perspectives of veterinary students enrolled in an international Spanish university regarding their knowledge, use, and perceived usefulness of AI tools. One of its key strengths lies in its scope and depth. The research included responses from 604 students of 34 nationalities, distributed across all courses within the veterinary degree program and coming from both secondary education and other higher education programs. The characteristics of the participants are an added benefit providing a diverse and globally informed viewpoint on the topic. Additionally, the demographic data show that the participants were a representative cohort of the veterinary students, as they are consistent with other studies regarding gender, age and future professional career preferences (51, 52). This investigation offers an extensive and detailed analysis with a design that facilitates a deeper understanding of veterinary students’ specific attitudes towards AI. IRT analysis showed a main cluster of highly interconnected items related to frequency of use and confidence, whereas a more peripheral strand connecting perceptions of AI’s professional consequences suggest that views on AI’s disruptive potential, though related to the main discussion, occupy a somewhat separate conceptual space. Furthermore, the factor analysis revealed three distinct areas of focus (confidence, education, and operational use and satisfaction) each representing a key dimension of students’ experiences with AI.

### 4.1 Use, competence and sources of AI information

AI is a set of systems that is gaining increasing interest in society in general, and in higher education in particular. More precisely, a rapid increase in the number of articles dealing with AI in veterinary medicine has been identified since 2019 (31). This is reflected in the interest expressed by participants in our sample, with only 3.4% saying they have never used AI and have no interest in doing so. On the other hand, 51% have already used AI, and 41% consider they know strengths and weaknesses of AI-assisted tools. A varying proportion of students indicated that they were unable to answer certain survey questions. The frequency of “I do not know” responses differed significantly across items, suggesting notable disparities in students’ familiarity or confidence. Notably, questions related to satisfaction with AI tools attracted the highest rates of non-response. In contrast, items focused on learning outcomes and concept clarification showed minimal uncertainty. Questions addressing confidence in AI fell somewhere in between. These patterns suggest that while students generally feel capable of assessing the educational value of AI, many hesitate to evaluate their satisfaction with specific AI applications, likely due to limited direct experience with these tools. Therefore, the survey reveals that more than 90% of the students have at least some knowledge and some practice with AI tools. These results indicate a somewhat higher usage than that reported in a similar study in Australia, which indicated use of ChatGPT by 77% of veterinary students (53). The high usage rate observed in our study may be partially attributed to potential selection bias in our sample. Additionally, another work on medical students in the United States found that about half of the students had used ChatGPT with more than 40% of users utilizing it almost weekly (54). Our study is not limited to the use of this tool, but in our case, the use of text-generating applications is clearly higher than other utilities, with only 6.3% of students reporting never having used it. Another research indicates an apparent lower use of AI, in this case by veterinary medicine students in Turkey (55). Other authors have described significant regional differences in use and in their perceptions of the usefulness and future of AI by medical, dental, and veterinary students (49). It is likely that cultural differences influence the results shown in these studies, but it is also conceivable that the higher percentage of users in our investigation reflects a rapid increase in the use of AI in higher education. This fact also suggests a growing interest among students, which is reflected in their demand for training, as will be discussed below. In fact, the study by Zhang et al. (54) shows that nearly half of ChatGPT users are more likely to use ChatGPT rather than directly asking professors or consulting textbooks. These data indicate the increasing adoption of AI tools for learning. Universities should identify the tools that best align with their educational objectives and actively prepare and train students to use them effectively and responsibly.

Despite the growing usage of certain AI tools, our analysis reveals that students exhibit moderate to low competence in areas such as AI regulation and the use of AI tools specific to the veterinary field. These findings are consistent with a previous study, which has shown that medical and health sciences students often have a limited knowledge of AI and its application in healthcare (56). Another study shows that half of dentistry students had basic knowledge of applied AI to this field (57).

There is partial agreement regarding students’ information sources. In our survey, the percentage of students who identified social networks as their primary source of information was similar to those who cited family and friends. However, Araujo and collaborators found that the media, including audiovisual and social networks, were the main source of information, a finding echoed by Singh et al. (56) among dental students. Moreover, in their case, the information received from both within and outside the university was very limited; however, in our case, nearly 70% of the students reported having received information from the faculty. These results suggest that despite the predominant use of AI in academic contexts, students are not primarily learning to use AI through their academic instruction. Therefore, there is significant space for improvement in integrating AI-related teaching into the curriculum to provide rigorous training in the use of these tools.

Altogether, the responses suggest that participants in our sample, while expressing positive attitudes, reported limited skills in certain areas. This is consistent with a systematic review that shows that healthcare students had a positive and promising attitude towards AI in medicine, but most of them had low knowledge and limited skills in working with AI (58).

### 4.2 Perceived potential impact of AI on the future of the profession

One of the risks associated with the rapid development of various AI tools is the potential threat to the continuity of certain professions. In particular, the advancement of technologies capable of quickly diagnosing conditions and proposing treatments has led to some uncertainty among students and professionals in the health sciences. In this regard, the opinions expressed in our study diverge from other research. Half of the participants in the survey by Civaner et al. (47) expressed concerns about the potential reduction in doctors’ roles, the devaluation of the medical profession, and possible harm to trust and the doctor-patient relationship. Similarly, 37.6% of medical students in the study by Jackson et al. (46) reported fears that AI might replace. However, in our study, positive perceptions of AI’s impact on the veterinary profession prevailed. Most participants in this study did not express concern that AI would reduce the usefulness of veterinarians and, on the contrary, a majority think it will enhance animal management and improve veterinary decision-making. These findings may reflect a generally positive outlook among participants regarding the role of AI in the veterinary profession and suggest an openness potential of these tools. It is likely that factors such as the different degrees studied (medicine vs. veterinary medicine) in different territories may be affecting the different perceptions that these studies show, but we cannot rule out that the time that has passed since the publication of both studies has changed the students’ approach to these issues. Even though the results of this work are close in time, the very rapid evolution of AI and its knowledge and use raises the possibility that the more it is used and known, the more possibilities for development are seen. This rapid evolution of AI technology makes it likely that it will be better accepted by younger generations. In fact, a survey on use of Chat-GPT for clinical practice and medical education, graduate physicians rated lower than medical students the use of this AI tool both for clinical practice and medical education (59).

### 4.3 University’s role in AI student training

Most participants in our sample agreed or strongly agreed that universities should provide training on AI, and, consequently, reject prohibiting the use of AI, but are in favor of regulating it. Similar opinions are found among veterinary students at the University of Sydney (53) and medical students in Pakistan (60). Additionally, an international survey of medical, dental, and veterinary students indicates that three-quarters of surveyed students would like to have more teaching on AI as part of their curricula (49).

Concerning technical issues, the areas in which training is most strongly desired by our students are those that allow improving the clinical follow-up of patients, but also in applications that facilitate the management of the clinic, probably because this subject is particularly arid for veterinarians. Similarly, health science students from various disciplines expressed the need for more training in tools that enhance practical skills, help solve medical problems, and reduce errors, as well as prevent and address ethical issues arising from the use of AI (47).

Gradually, universities are integrating AI-related tools into their subjects. Text-generating AIs have been proposed for various applications in veterinary education, including extracting patient data, generating progress notes, assisting in complex diagnoses, supporting student learning, and aiding in exam preparation and academic writing. Additionally, veterinary educators can create custom GPTs for tailored student support (36, 37). A specific example of an AI-driven activity in equine medicine is the work of Sousa and Flay, in which students created multiple-choice questions and evaluated ChatGPT’s responses. In it, most students found the activity engaging and felt it helped deepen their understanding and critical thinking skills (61). That study also shows that students with previous AI experience showed higher levels of engagement and perceived the exercise as more effective, suggesting that increasing AI training can improve their use of AI and thus their learning.

Furthermore, it should be noted that these tools are beginning to show effectiveness in clinical settings. Indeed, text generation tools such as ChatGPT, Gemini, or Copilot can have diagnostic utility, although with different efficiency (62). For example, in human psychiatry, it has been shown that ChatGPT has appreciable knowledge and interpretation skills (63). Furthermore, the relative efficacy of large language models (LLMs) such as ChatGPT has been tested in the diagnosis of eye problems (64) and in medical decision-making in infectious diseases (65).

AI-based tools for professional use, particularly those related to image diagnosis, are noteworthy. Significant developments have been made in conventional radiology, ultrasound, computed tomography, and magnetic resonance imaging, with some AI-based programs still at an experimental stage. These programs assist veterinarians in diagnosis (19) and rapid advancements in these systems are expected soon. Nevertheless, other areas of the veterinary profession are experiencing the advent of AI-based tools. For instance, AI software is already beginning to be developed for early detection of ocular bovine pathologies (21) or ruminant livestock management (66). Universities should therefore be aware of this situation and ensure that students receive adequate training in these tools.

The usefulness of AI-based technologies in higher education will depend on how their development is implemented and how students feel they can own and trust them. Several key factors have been described to influence students’ adoption of ChatGPT and similar AI tools for learning and research. Interactive and collaborative learning are crucial for student engagement. Students are more likely to adopt AI if it improves their engagement and academic performance. Trust in the accuracy and reliability of AI-generated content, as well as the perceived ease of use and usefulness of the tools, is also important (48). In this sense, it seems particularly important to teach students to use AI as tools for exploring topics, but also to emphasize the need to validate the information they provide. In this regard, experiences have been described where students use LLMs to generate essays and then analyze the AI-generated sources and the validity of the scientific claims, based on experimental evidence from primary sources (67). Interestingly, students are sometimes using generative AI in innovative ways to enhance their learning and personal lives in areas as diverse as academic preparation, language learning or as a personal assistant. The use of chatbots and generative AI tools is transforming the education and personal lives of students, offering new ways of learning and organising that complement their studies and daily activities (68).

### 4.4 Ethical considerations in the development and use of AI in higher education

To what extent is it advisable to use AI in university studies? In this regard, there are differences in the positions of universities concerning the use of AI in their classrooms. As examples, some policies related to the use of AI in different subjects at different universities have been proposed (69). It seems that the rapid development of AI applications has made it challenging for higher education institutions to adapt to the ways students might use these tools, resulting in significant heterogeneity in terms of permissiveness. We believe that universities cannot ignore the existence of generative AI and, as institutions dedicated to higher education, they must consider both the technical and ethical training needs of their students.

On the other hand, teachers often do not know how to address the ethical challenges posed using AI. This lack of awareness underscores the need for a comprehensive understanding of the ethical implications of AI, especially as its integration into educational environments and professional fields continues to expand, raising critical questions about responsibility, fairness and privacy. In this sense, a study revealed varied and often conflicting views on AI ethics among educators, highlighting a general lack of awareness and inconsistent application of ethical principles, as well as challenges in understanding and managing ethical issues related to AI (70).

In the same way, it is crucial to acknowledge the limitations of AI and to use it ethically. Institutions should develop guidelines that reflect their unique educational values, offering clear frameworks to support responsible use of LLMs while addressing the associated risks in education, as it has been described (71).

Additionally, we cannot forget that many educators lack sufficient training to adequately prepare students. Addressing these demands requires institutional support and possibly a shift in pedagogy to ensure a sustainable and effective integration of AI, as has already been suggested by other authors in the case of medical education (72). In this context, it has been proposed that educational institutions organize regular workshops to show how AI tools can be practically integrated into academic activities, highlighting their benefits to reduce perceived complexity and increase confidence in using these tools. Additionally, AI developers can also produce tools tailored to academic contexts, addressing specific needs of teachers (73).

Understanding these factors is essential for educators and policymakers seeking to optimize AI integration in veterinary education and beyond, ensuring that technological advancements align with students’ needs and expectations.

### 4.5 Limitations of the study

The study relies on self-reported data, which may introduce biases or inaccurate self-assessment by the students. The use of self-reported data may introduce social desirability effects, as students might have provided answers they perceived as favorable or expected. Moreover, selection bias may be present, as participation was voluntary and students with a stronger interest in AI and a higher level of academic engagement may have been more likely to respond. This may have contributed to an overestimation of positive attitudes and reported usage rates of AI tools among the respondents. This is a single-center study, so the generalization of the results presented here should be approached with caution. It should be noted that including students from other institutions could yield different results. However, the diverse backgrounds and nationalities of the participants, as well as the high number of responses obtained, allow us to consider that our results may represent a general overview of the topics addressed. Finally, since this study focuses on students’ perceptions of their relationship with AI tools, objective metrics for measuring student learning are not used. This analysis goes beyond the scope of this study and may be examined in future research.

## 5 Conclusion

This study provides a detailed snapshot of the perceptions on AI of a large, multinational cohort of students enrolled at a single Faculty of Veterinary Medicine. With over 600 participants from 34 nationalities and all academic years, it provides a diverse perspective on the integration of AI in veterinary education. The findings indicate a high frequency of AI tool usage, particularly generative tools like ChatGPT. Participants in our study predominantly reported using AI in academic contexts and described it as a valuable educational tool, particularly for synthesizing scientific information. However, they consider additional training is needed in veterinary-specific AI tools. Participants expressed strong interest in the integration of AI-related education within the veterinary curriculum and reported a desire to learn more about its applications dedicated to various veterinary fields. Furthermore, the study reveals a significant concern regarding the regulation of AI use and reliability of AI responses. These issues highlight the importance of integrating ethical considerations and verification processes to be integrated into AI-related education.

Together, our findings underscore the potential value of targeted educational strategies that not only build confidence and familiarity with AI tools but also address foundational issues such as transparency, ethics, and governance. By providing targeted training and support, universities can help veterinary students develop the skills and knowledge needed to harness the potential of AI while avoiding the pitfalls of its misuse in their professional careers.

## Data Availability

The raw data supporting the conclusions of this article will be made available by the authors, without undue reservation.
