# Supplementary material for: Integrating artificial intelligence into veterinary education: student perspectives
Source: Front Vet Sci. 2025 Aug 4;12:1641685. doi: 10.3389/fvets.2025.1641685 (PMC12358847; doi:10.3389/fvets.2025.1641685)
Supplement: Supplementary file 1 [file Data_Sheet_1.pdf]

Supplementary data 1: Survey

Idioma: English (United Kingdom)

Multilingual questionnaire, pilot project

Information for the participants Title of the project: *Perception and Use of Artificial Intelligence by Veterinary Students: A Pilot Study* **Principal investigator:** Christelle de Brito - University CEU Cardenal Herrera. We are contacting you to inform you about a research study in which you are invited to participate. Our intention is to provide you with the correct and sufficient information so that you can decide if you accept to participate in this study. If you decide to participate, you can keep a copy of this document by sending the questionnaire. The study has been approved by the Research Ethics Committee of the University CEU Cardenal Herrera in Valencia. It has been designed and will be conducted in accordance with the recommendations established in the Declaration of Helsinki. You should know that your participation in this study is **anonymous** and **voluntary**, and you can decide **NOT** to participate. Participation or non-participation will not alter your relationship with your professors.

WHY IS THIS STUDY BEING CONDUCTED? Artificial intelligence (AI) has been established as a potentially very powerful tool in veterinary education and profession. Therefore, this project aims to study the training on AI, as well as the use and perception of AI by veterinary students.

HOW WILL THE STUDY BE CONDUCTED? This project is based on the analysis of responses to the anonymous and voluntary questionnaire below. The questionnaire will be available for a period of 2 months from the start of its dissemination.

WHAT CRITERIA MUST BE MET TO PARTICIPATE? Participants must be students enrolled in the Faculty of Veterinary Medicine at the University CEU Cardenal Herrera.

WHAT DOES MY PARTICIPATION INVOLVE? Participation is voluntary and consists of completing the anonymous questionnaire. It does not involve any intervention or risk.

WHAT BENEFITS WILL BE OBTAINED FROM THE STUDY? It is expected that this project will provide conclusions on the training, use, and perception of AI by veterinary students, which should facilitate the design of future training activities in veterinary education.

HOW ARE MY RIGHTS PROTECTED? The study will be conducted in compliance with all current ethical and legal standards. Data will be collected anonymously in a research file managed by the researchers and will be processed on computers of the university net that can only be accessed with a personal password. The collected data will never be used for any other purpose, and all data will be deleted once the research results have been published.

WHO CAN I CONTACT IF I HAVE QUESTIONS? If you have any questions, please contact Christelle de Brito (University CEU Cardenal Herrera): christelle.debrito@uchceu.es.

Whatever your decision is, the research team would like to thank you for your time and attention.

* Obligatoria

Information about the participant

1. I am enrolled in *

- ◯ 1st year of Veterinary Medicine of the University CEU Cardenal Herrera
- ◯ 2nd year of the Veterinary Medicine of the University CEU Cardenal Herrera
- ◯ 3rd year of Veterinary Medicine of the University CEU Cardenal Herrera
- ◯ 4th year of Veterinary Medicine of the University CEU Cardenal Herrera
- ◯ 5th year of Veterinary Medicine of the University CEU Cardenal Herrera
- ◯ None of the previous proposals

2. Answer the option that best applies to you, in relation to artificial intelligence (AI) *

- ◯ I have never used artificial intelligence-assisted tools and I have no interest in artificial intelligence (AI) in general
- ◯ I am interested in artificial intelligence (AI) but I have never used artificial intelligence-assisted tools
- ◯ I have used an AI-assisted tool at least once in my life
- ◯ I believe I have knowledge about the strengths and weaknesses of AI-assisted tools

3. Year of birth *

Enter a number greater than 1980.

4. Gender *

- Male
- Female
- I prefer not to answer

5. Nationality *

- Spanish
- French
- Other

6. Nationality *

7. Indicate the **higher education** studies you have completed **before** joining the Faculty of Veterinary Medicine *

- ◯ I have not completed higher education studies before joining the Faculty of Veterinary Medicine
- ◯ Bachelor’s Degree in Sciences
- ◯ Engineering Studies
- ◯ Higher Technician Degree
- ◯ Other

8. Which veterinary specialty interests you the most? *

- ◯ Clinic (prevention, diagnosis, treatment, rehabilitation, etc.)
- ◯ Health and Public Health (hygiene, food inspection, agro-food, etc.)
- ◯ Animal Production (reproduction, production, etc.)
- ◯ Conservation of Wildlife and the Environment
- ◯ Other

Training in Artificial Intelligence

9. Indicate your level of agreement with the following statements *

	Strongly disagree	Somewhat disagree	Neutral	Somewhat agree	Strongly agree	I don't know
AI facilitates obtaining clear explanations about complex concepts in Veterinary Medicine courses	○	○	○	○	○	○
AI generates questions that help with exam preparation	○	○	○	○	○	○
AI allows summarizing my Veterinary Medicine courses	○	○	○	○	○	○
AI allows generating clinical cases and patient simulations	○	○	○	○	○	○

10. In what context have you most used AI tools?*

- ◯ In the academic context
- ◯ In a non-academic context

11. Please indicate the **level of proficiency** you believe you currently have regarding: *

	None	Low	Moderate	High	Very High
The principles of AI operation	◯	◯	◯	◯	◯
The regulation of AI	◯	◯	◯	◯	◯
The use of AI-assisted tools **in general**	◯	◯	◯	◯	◯
The use of AI-assisted tools in **the veterinary field**	◯	◯	◯	◯	◯

12. To what extent have you acquired these skills in artificial intelligence thanks to the teaching at the Faculty of Veterinary Medicine? *

- Not at all
- A little
- Moderately
- A lot
- Completely

13. By what means did you obtain the most practical information about AI? *

- Faculty of Veterinary Medicine
- Education studies completed before joining the Faculty of Veterinary Medicine
- Friends or Family
- Magazines
- Videos
- Social media
- Other

14. Indicate your level of agreement with the following statements *

	Strongly disagree	Somewhat disagree	Neutral	Somewhat agree	Strongly agree
Veterinary Faculties should encourage the use of AI by students	○	○	○	○	○
Veterinary Faculties should train their students in the use of AI	○	○	○	○	○
Veterinary Faculties should regulate the use of AI by students	○	○	○	○	○
The use of AI during training should be prohibited	○	○	○	○	○

15. Which aspects linked to the AI would you like to cover during your training at the Faculty of Veterinary Medicine (you can select **multiple** options)? *

- [ ] Ethical and regulatory issues
- [ ] Applications aimed at assisting a veterinarian's decision-making
- [ ] Applications aimed at assisting the clinical follow-up of patients
- [ ] Applications aimed at assisting the follow-up of production animals
- [ ] Applications aimed at assisting the follow-up of wildlife
- [ ] Applications aimed at assisting in epidemic tracking
- [ ] Applications aimed at assisting scientific research
- [ ] Applications aimed at assisting the management of a veterinary clinic
- [ ] I do not wish to address these topics during my training

Use of artificial intelligence

16. Indicate **how often** you use these tools in relation to your studies at the Faculty of Veterinary Medicine *

	never	less than once a month	1 to 3 times a month	1 to 3 times a week	more than 3 times a week
Text generator (ChatGPT, Copilot...)	○	○	○	○	○
Image analysis for diagnostic imaging	○	○	○	○	○
Tools to assist in clinic management	○	○	○	○	○
Clinical case simulator	○	○	○	○	○
Bibliography writing assistant (Zotero, Mendeley...)	○	○	○	○	○

17. What is your **level of satisfaction** with the tools you use (if you do not use them, check "I don’t know")? *

	Not satisfied at all	Slightly satisfied	Satisfied	Very satisfied	Extremely satisfied	I don’t know
Text generator (ChatGPT, Copilot...)	◯	◯	◯	◯	◯	◯
Image analysis for diagnostic imaging	◯	◯	◯	◯	◯	◯
Tools to assist in clinic management	◯	◯	◯	◯	◯	◯
Clinical case simulator	◯	◯	◯	◯	◯	◯
Bibliography writing assistant (Zotero, Mendeley...)	◯	◯	◯	◯	◯	◯

18. Which tools do you find **most promising** in the field of veterinary medicine?

Perception of artificial intelligence

19. Indicate your **level of confidence** regarding the following elements: *

	none	low	neutral	high	very high	I don't know
AI-generated clinical cases	○	○	○	○	○	○
Veterinary diagnosis established by AI	○	○	○	○	○	○
Veterinary treatment recommended by AI	○	○	○	○	○	○
Synthesis of scientific information performed by AI	○	○	○	○	○	○
Confidentiality of personal data processing by AI	○	○	○	○	○	○

20. Indicate your level of agreement with the following statements *

	Strongly disagree	Somewhat disagree	Neutral	Somewhat agree	Strongly agree
AI-provided responses should be verified	○	○	○	○	○
I would like to be informed when a tool is assisted by AI	○	○	○	○	○
I would like information to be available about how AI-assisted tools work, particularly about the algorithms used	○	○	○	○	○
Human decisions should prevail over recommendations given by an AI-assisted tool	○	○	○	○	○

21. Indicate your level of agreement with the following statements *

	Strongly disagree	Somewhat disagree	Neutral	Somewhat agree	Strongly agree
AI will have a great impact on the veterinary profession in general	○	○	○	○	○
AI will decrease the usefulness of veterinarians	○	○	○	○	○
AI will improve the cares of animal patients	○	○	○	○	○
AI will improve decisions made by veterinarians	○	○	○	○	○
AI will make some veterinary specialties disappear	○	○	○	○	○
AI will reduce the workload of veterinarians	○	○	○	○	○

When I become a veterinarian, will frequently use AI-assisted tools	○	○	○	○	○

22.. Optional: you can leave here comments on the use of AI in an academic activity.

Este contenido no está creado ni respaldado por Microsoft. Los datos que envíe se enviarán al propietario del formulario.

Microsoft Forms
